# The calcium-binding protein S100P in normal and malignant human tissues

**DOI:** 10.1186/1472-6890-8-2

**Published:** 2008-02-18

**Authors:** Seppo Parkkila, Pei-wen Pan, Aoife Ward, Adriana Gibadulinova, Ingrid Oveckova, Silvia Pastorekova, Jaromir Pastorek, Alejandra Rodriguez Martinez, Henrik O Helin, Jorma Isola

**Affiliations:** 1 Institute of Medical Technology, University of Tampere and Tampere University Hospital, Tampere, Finland; 2 School of Medicine, University of Tampere and Tampere University Hospital, Tampere, Finland; 3 Institute of Virology, Slovak Academy of Sciences, Bratislava, Slovak Republic

## Abstract

**Background:**

S100P is a Ca^2+ ^binding protein overexpressed in a variety of cancers, and thus, has been considered a potential tumor biomarker. Very little has been studied about its normal expression and functions.

**Methods:**

We examined S100P expression in normal human tissues by quantitative reverse transcription polymerase chain reaction and immunohistochemistry. S100P protein expression was also studied in a series of tumors, consisting of 74 ovarian, 11 pancreatic, 56 gastric, 57 colorectal, 89 breast and 193 prostate carcinomas using a novel anti-S100P monoclonal antibody.

**Results:**

Among the normal tissues, the highest S100P mRNA levels were observed in the placenta and esophagus. Moderate signals were also detected in the stomach, duodenum, large intestine, prostate and leukocytes. At the protein level, the highest reactions for S100P were seen in the placenta and stomach. Immunostaining of tumor specimens showed that S100P protein is expressed in all the tumor categories included in the study, being most prevalent in gastric tumors.

**Conclusion:**

Based on our observations, S100P is widely expressed in both normal and malignant tissues. The high expression in some tumors suggests that it may represent a potential target molecule for future diagnostic and therapeutic applications.

## Background

The S100 proteins belong to the EF-hand superfamily of Ca^2+ ^binding proteins that mediate Ca^2+ ^dependent signal transduction pathways involved in the regulation of cell cycle, growth, differentiation and metabolism [1]. S100 proteins have been functionally associated with various neurological, cardiac and neoplastic diseases.

S100P protein is a relatively small (95 amino acid) isoform of the S100 protein family that was first isolated from human placenta [2]. Overexpression of S100P has been detected in several cancers such as breast [3], colon [4], prostate [5], pancreatic [6] and lung [7] carcinomas, and the protein has been functionally implicated in carcinogenic processes [8-10]. In pancreatic cancer, S100P is overexpressed due to hypomethylation of its gene [11]. Studies on prostate cancer have indicated that S100P expression is regulated by androgens [5] and interleukin-6 [12]. In gastric cancer cell lines, retinoic acid has been reported to induce S100P expression [13]. In breast cancer cell lines, S100P overexpression seems to be an early event that has been suggested to play a role in the immortalization of human breast epithelial cells *in vitro *and tumor progression *in vivo *[3]. In colon cancer cell lines, expression level of S100P correlated with resistance to chemotherapy [14], and in lung and breast cancer to decreased patient survival [7,15]. However, despite these observations, little is still known about the functional role or mechanism of action of S100P. Recently, it has been shown that S100P can induce anchorage-independence of tumor cells *in vitro *and improve tumor growth in a xenograft model. These results suggested that S100P functionally participates in the control of the tumorigenic potential *in vivo *[9].

In the present study, we describe a novel monoclonal antibody for S100P protein designated 18-9 and evaluate S100P expression in normal and neoplastic human tissues by immunohistochemistry and quantitative reverse transcription-polymerase chain reaction (RT-PCR). This data could provide valuable information on where S100P is expressed under normal and pathological conditions, and whether it could serve as a tissue- or tumor-specific biomarker.

## Methods

### Quantitative real-time PCR

The amount of human S100P transcript in different tissues was assessed by quantitative real-time RT-PCR using the Lightcycler detection system (Roche, Rotkreuz, Switzerland). Real-time PCR primers were designed on the basis of the complete cDNA sequences deposited in GenBank (accession number: NM_005980). The primers were located in two different exons separated by a 2822 bp-long intron. The sequences were as follows: forward primer: 5'-TCAAGGTGCTGATGGAGAA-3', reverse primer: 5'-ACACGATGAACTCACTGAA-3'. Three housekeeping genes (YWHAZ: Tyrosine 3-monooxygenase/tryptophan 5-monooxygenase activation protein, zeta polypeptide, GAPD: Glyceraldehyde-3-phosphate dehydrogenase, and UBC: Ubiquitin C) were used as internal RNA controls to normalize the cDNA samples for differences [16]. The templates for the PCR reactions were obtained from cDNA kits (human MTC™ digestive panel, panel I, panel II and blood fractions panel) purchased from BD Biosciences (Palo Alto, CA). These kits contained first-strand cDNA preparations produced from poly(A) RNAs isolated from different organs and cell fractions. The numbers of pooled tissue specimens for each RNA sample were as follows: placenta (n = 7), spleen (n = 11), thymus (n = 18), prostate (n = 32), testis (n = 45), ovary (n = 5), leukocyte (n = 550), ascending colon (n = 5), descending colon (n = 7), transverse colon (n = 19), duodenum (n = 30), ileocecum (n = 19), ileum (n = 8), jejunum (n = 6), rectum (n = 6), cecum (n = 29), stomach (n = 7), esophagus (n = 39), mononuclear cells (n = 12), resting CD8+ cells (n = 20), resting CD4+ cells (n = 11), resting CD14+ cells (n = 36), resting CD19+ cells (pooled from Caucasian blood donors, number not provided), activated CD19+ cells (n = 4), activated mononuclear cells (n = 4), activated CD4+ cells (n = 6) and activated CD8+ cells (n = 8).

Every PCR was performed in a total reaction volume of 20 μl containing 0.5 μl of first strand cDNA, 1× of QuantiTect SYBR Green PCR Master Mix (Qiagen, Hilden, Germany), and 0.5 μM of each primer. Amplification and detection were carried out as follows: After an initial 15-min activation step at 95°C, amplification was performed in a three-step cycling procedure: denaturation at 95°C, 15 s, ramp rate 20°C/s; annealing at the temperature determined according to the melting temperature for each primer pair (52°C for S100P, 59°C for YWHAZ and GADP, and 57°C for UBC), 20 s, ramp rate 20°C/s; and elongation at 72°C, 20 s, ramp rate 20°C/s for 45 cycles and final cooling step. The melting curve analysis was always performed after the amplification to check PCR specificity. To quantify the levels of transcripts for reference genes and S100P in tissue specimens, a standard curve for each gene was established using five-fold serial dilutions of known concentrations of purified PCR products generated from the same primer sets. Each cDNA sample was tested in duplicate, and the crossing point (Cp) value obtained allowed us to determine the amount of the starting message using the specific standard curve. The geometric mean of the three reference genes was used as a normalization factor for gene expression levels [16]. The copy number of S100P in each tissue was divided by the corresponding normalization factor and subsequently multiplied by 10^2^.

### Antibodies

The monoclonal anti-human S100P antibody 18-9 was produced by hybridoma technology as follows: Six weeks old BALB/c mice were immunized by two i.p. inoculations of GST-S100P fusion protein purified from bacteria transfected with pGEX-3X-S100P plasmid. The plasmid contained the full length S100P cDNA subcloned from the pSG5C-S100P eukaryotic vector described earlier [9]. The first immunization dose consisted of 50 μg GST-S100P in 250 μl phosphate-buffered saline (PBS) and 250 μl Titer Max Gold adjuvant (Sigma). Three weeks later, the mice received a challenge in the form of 250 μl suspension of GST-S100P protein bound to the Glutathione-S Sepharose in absence of adjuvant. The splenocytes were harvested after two weeks and fused with the Sp2/0 myeloma cells. The obtained hybridomas were screened by ELISA using GST-S100P fusion antigen versus GST alone. Specific reactivity of the produced monoclonal antibodies was verified by Western blotting using the cell lines naturally or ectopically expressing S100P. The best hybridomas were subcloned and frozen. The hybridoma clone 18-9 was expanded and the MAb-containing hybridoma medium was used for further studies. Another monoclonal S100P antibody (control MAb) was purchased from BD Biosciences Pharmingen (San Diego, CA).

### Western blotting

Cells grown in confluent monolayer were rinsed twice with cold PBS and solubilised in ice-cold RIPA buffer (1% Triton X-100 and 1% deoxycholate in PBS) containing the commercial COMPLETE cocktail of protease inhibitors (Roche Diagnostics GmbH, Mannheim, Germany) for 30 min on ice. The extracts were collected, cleared by centrifugation at 15 000 rpm for 10 min at 4°C and stored at -80°C. Protein concentrations of the extracts were quantified using the BCA protein assay reagent (Pierce, Rockford, IL).

The extracts were resolved in 12% SDS-PAGE and transferred to PVDF membrane (Amersham Pharmacia Biotech, Little Chalfont Buckinghamshire, UK). After blocking in 5% non-fat dry milk with 0.2% Nonidet P40 in PBS, the membrane was probed with the MAb 18-9, washed and treated with secondary anti-mouse HRP-conjugated swine antibody diluted 1/7500 (Sevapharma, Prague, Czech Republic). The protein bands were visualized by enhanced chemiluminescence using the ECL kit (Amersham Pharmacia Biotech).

### Reverse transcription PCR

Total RNA was isolated from the cell monolayers using the INSTAPURE solution (Eurogentech, Belgium) according to the protocol of the manufacturer. Reverse transcription was performed with Mo-MuLV reverse transcriptase (Finnzymes OY, Espoo, Finland) as described before [9]. RT-PCR was performed with Taq DNA polymerase (Finnzymes) in an automatic DNA thermal cycler (Eppendorf AG, Hamburg, Germany). Following an initial denaturation at 95°C for 3 min, the amplification program was set as follows: denaturation at 95°C for 30 sec, annealing at 60°C for 30 sec, and extension at 72°C during 40 sec for a total of 30 cycles, and finally 5 min at 72°C. Resulting PCR fragments were run on 2% agarose gels. The nucleotide sequences of the primers were as follows (s, sense; a, antisense): S100P-s (109-130) 5'-AAGGGGGAGCTCAAGGTGCTGA-3', S100P-a (330-308) 5'-ATCTGTGACATC TCCAGGGCATC-3', S100A4-s 5'-GCAAAGAGGGTGACAAGTTCAAG-3', S100A4-a 5'-GATGCAGGACAGGAAGACACAGT-3'.

### Immunohistochemistry

The normal tissue and tumor specimens were collected at Oulu and Tampere University Hospitals. The tumor materials included 74 ovarian, 11 pancreatic, 56 gastric, 57 colorectal, 89 breast and 193 prostate carcinomas. All tissue samples were obtained during surgery according to the guidelines of the Declaration of Helsinki and processed for routine histopathological evaluation. Collection of specimens was approved by the local ethics committees. The specimens were first fixed in 4% neutral-buffered formaldehyde, dehydrated in ethanol series, treated with xylene and mounted in paraffin.

Automated immunostaining for S100P was performed using Power Vision+™ Poly-HRP IHC Kit reagents (ImmunoVision Technologies, Co.). The immunostaining method included the following steps: (a) deparaffinization of the sections using xylene and ethanol series; (b) treatment with Tris-EDTA buffer, pH 9.0, at 105°C for 15 min; (c) rinsing in wash buffer (Tris-buffered saline, pH 7.6, containing 0.05% Tween-20); (d) incubation with 18-9 monoclonal anti-S100P antibodies diluted 1:20 in Universal IHC Blocking/Diluent for 30 min; (e) rinsing in wash buffer; (f) blocking with Universal IHC Blocking/Diluent for 20 min; (g) rinsing in wash buffer; (h) incubation in Poly-HRP-conjugated anti-rabbit/mouse IgG for 30 min and rinsing in wash buffer; (i) incubation in DAB (3,3'-diaminobenzidine tetrahydrochloride) solution (one drop DAB solution A and one drop DAB solution B with 1 ml ddH_2_O) for 5 min; (j) rinsing with ddH_2_O; (k) 0.5% CuSO_4 _treatment for 5 min to enhance the signal; (l) rinsing with wash buffer; (m) counterstaining with hematoxylin solution for 2 min; and (n) rinsing with ddH_2_O. All procedures except for the step (b) were carried out at room temperature. The sections were mounted in Entellan Neu (Merck; Darmstadt, Germany) and finally examined and photographed with a Zeiss Axioskop 40 microscope (Carl Zeiss; Göttingen, Germany).

## Results

### S100P mRNA expression

**Figure 1:**
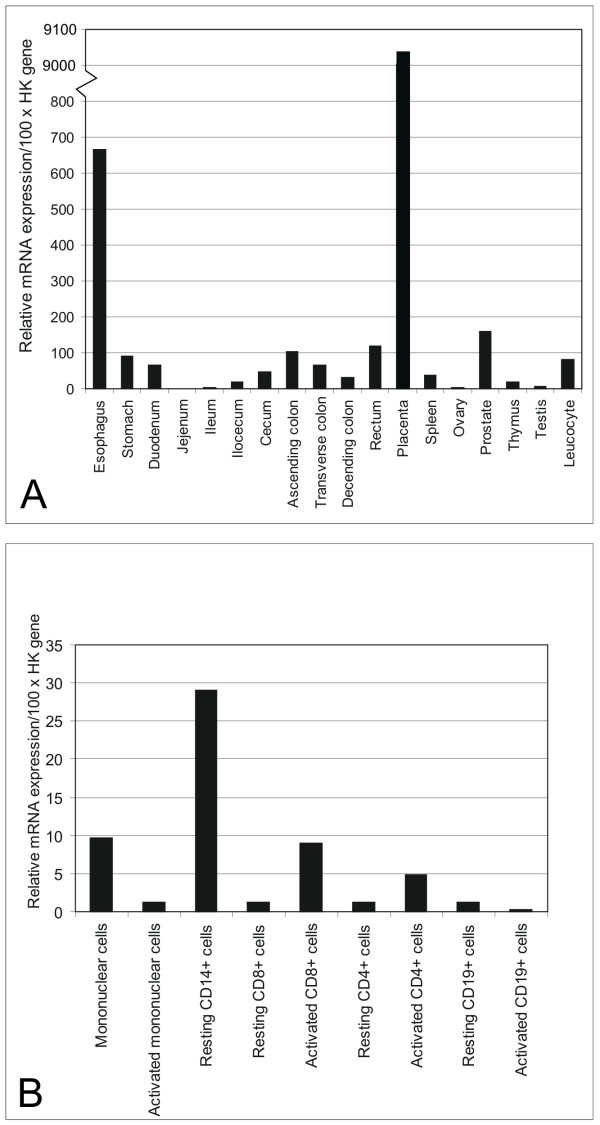
Relative S100P mRNA expression levels in normal human tissues and leukocyte fractions. Value 100 indicates the geometric mean of the expression levels of three housekeeping genes used in the study.

In our study, we first performed quantitative RT-PCR to analyze S100P mRNA levels in various normal human tissues. The PCR product from placenta was sequenced in order to confirm its identity and to exclude any unspecific binding of primers (data not shown). Figure 1A demonstrates that positive signals for S100P mRNA were detectable in most tissues. The highest expression levels were found in the placenta (~90.4 times the housekeeping gene expression levels). The esophagus also showed relatively high expression (~6.7 times the housekeeping gene expression levels). Other segments of the gastrointestinal canal including the stomach, duodenum and large intestine showed moderate mRNA levels which were lower or slightly higher compared to the housekeeping genes. The jejunum and ileum demonstrated almost negligible levels of expression. From the reproductive tract organs the prostate was moderately positive, whereas both ovary and testis displayed extremely low signals. Lymphatic tissues including the spleen and thymus as well as leukocytes were positive, but the expression levels remained lower compared to the housekeeping genes. We further analyzed different fractions of the human leukocytes for S100P expression (Fig 1B). The highest mRNA expression was detected in resting CD14+ cells representing the monocyte fraction of the blood cells. Unstimulated T-cells (CD4+ and CD8+) exhibited very weak signals which became slightly higher in activated cells.

### S100P protein expression

**Figure 2:**
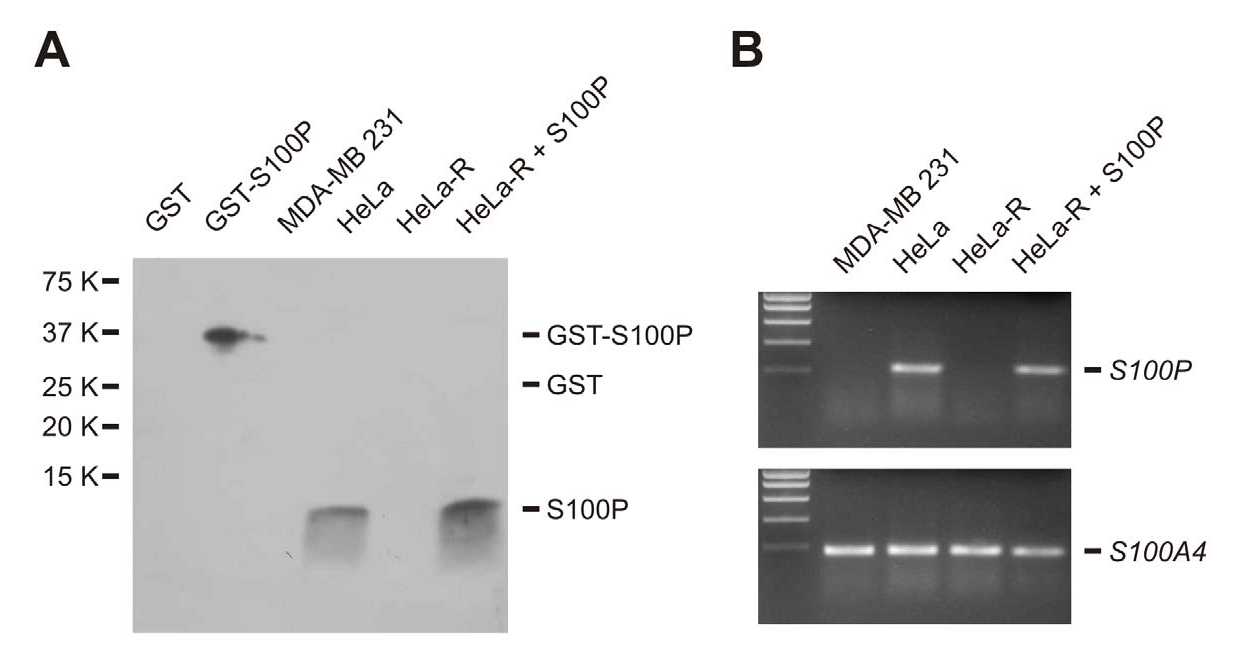
Specific detection of S100P with 18-9 MAb. **A**, Western blotting analysis of the recombinant GST-S100P protein versus GST alone and of S100P protein in extracts from MDA-MB 231 breast carcinoma cells, HeLa cervical carcinoma cells, HeLa-R variant cells with reduced tumorigenicity [9], and HeLa-R cells transfected with S100P cDNA. **B**, RT PCR analysis of the same cell lines for the expression of S100P and S100A4 genes, respectively.

In addition to the mRNA analysis, we wanted to elucidate S100P protein expression by immunohistochemistry in various human tissues. For this purpose, we used a newly generated anti-S100P monoclonal antibody 18-9 produced against a recombinant fusion GST-S100P antigen. First, we verified the specificity of the MAb (Fig. 2). In Western blotting, 18-9 MAb reacted well with the purified GST-S100P protein, but not with the GST alone. The MAb also recognized an 11 kDa protein in HeLa cells and HeLa-R cells transfected with S100P cDNA, whereas no protein was detected in MDA-MB 231 cells and mock-transfected HeLa-R cells (Fig. 2A). These negative cell lines do not express S100P, but show expression of S100A4, as described previously [9,15,17] and also clearly demonstrated in Figure 2B by RT PCR.

**Figure 3:**
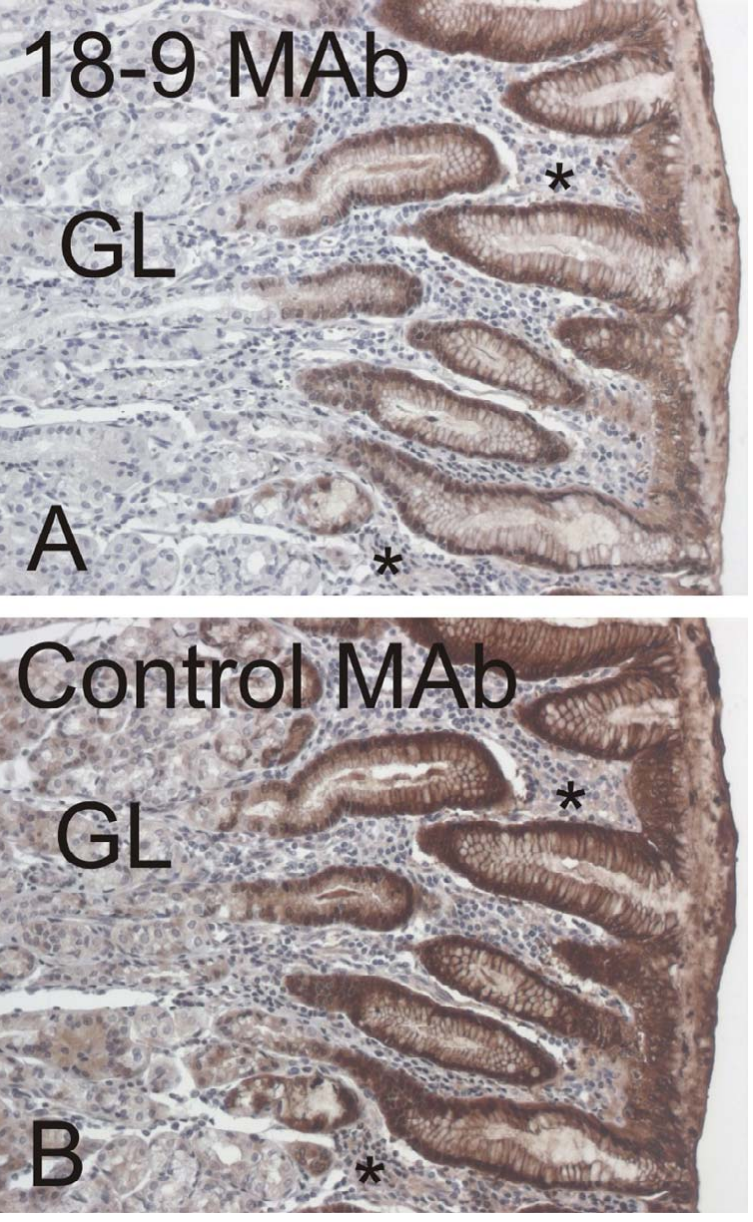
Immunohistochemical comparison of the reactivity of 18-9 MAb (A) versus control MAb (B) in parallel sections of the gastric antrum. In both panels, the highest immunoreactivity is located in the surface epithelial cells. Control MAb shows slightly higher staining in the glands (GL) and lamina propria (asterisk).

**Figure 4:**
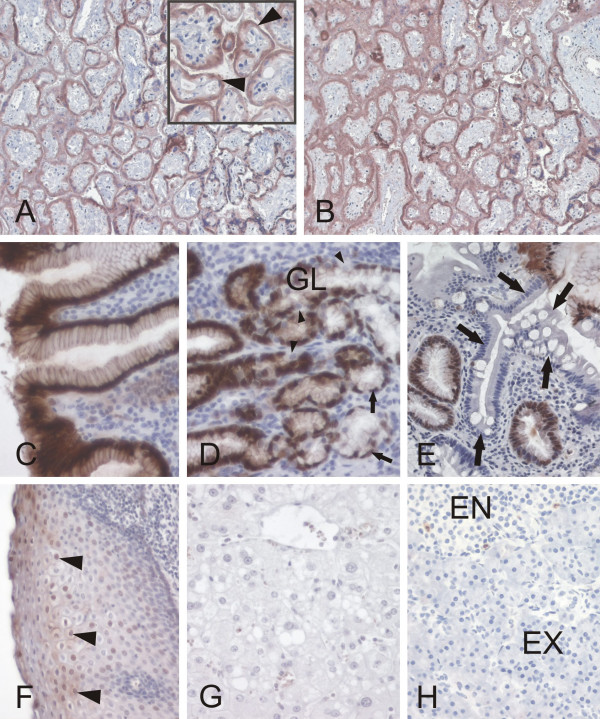
Immunohistochemical localization of S100P protein in normal human tissues. Panel A shows the staining reaction obtained by new 18-9 MAb in the placenta. Commercial control S100P antibody has been used in the panel B. Both antibodies immunostain trophoblast cells (arrowheads in the inset) lining the placental villi. Other panels demonstrate the distribution of S100P protein in the human gastric mucosa (C,D,E), esophagus (F), liver (G) and pancreas (H). The staining reactions are strong in the stomach, distinctly weaker in the esophagus and negative in the liver and pancreas. In the stomach, the most prominent signals were located to the mucus secreting epithelial cells. Positive reactions were also present in the principal cells (small arrows) of the gastric glands (GL). The parietal cells were negative or only weakly stained (small arrowheads). The large arrows in panel E point to the intestinal metaplasia that was occasionally seen in some specimens as a benign pathological change within the gastric mucosa. In panel F, the arrowheads indicate some positive nuclei in the esophageal epithelium. EN = endocrine pancreas, EX = exocrine pancreas.

**Figure 5:**
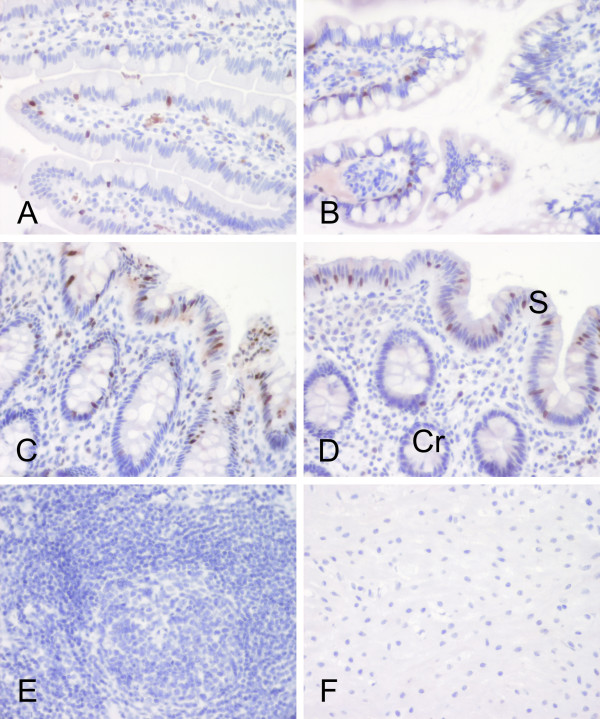
Localization of S100P protein in the human gut. All segments including duodenum (A), ileum (B), cecum (C) and colon (D) showed positive reactions in the enterocytes. In the large intestine, the most prominent reactions were observed in the surface epithelial cuff region (S). The reactions became weaker toward the crypts (Cr). Both the lymphoid follicles (E) and smooth muscle (F) of the intestinal wall remained negative.

**Figure 6:**
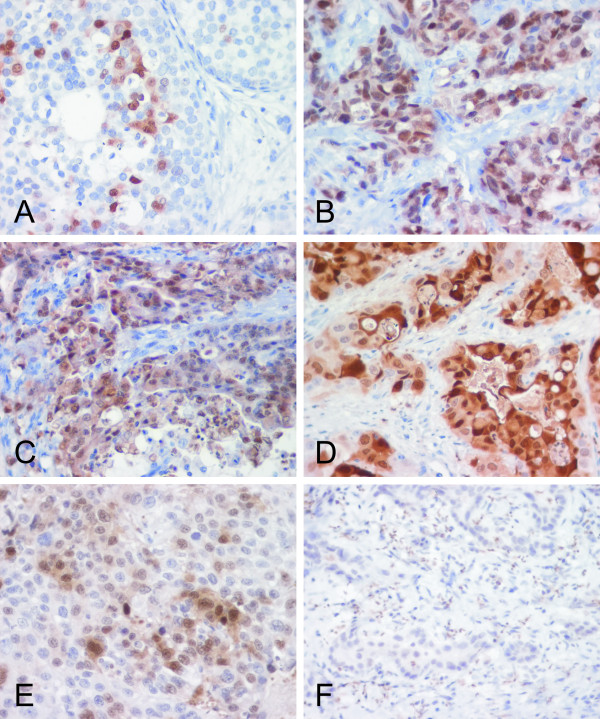
Immunostaining of S100P protein in the breast (A,B), gastric (C), pancreatic (D) and ovarian (E,F) carcinomas.

**Figure 7:**
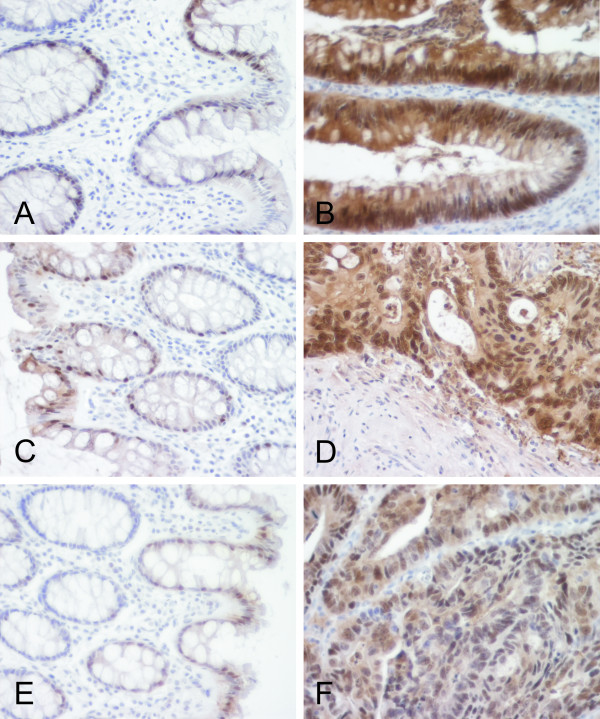
Distribution of S100P protein in a sample of colonic adenoma with moderate dysplasia (B), two colon carcinomas (D,F) and adjacent benign tissues (A,C,E). The protein is overexpressed in both benign and malignant tumors compared to the normal colonic mucosa.

The specificity of the 18-9 MAb was also evaluated by immunohistochemistry in comparison with the control MAb in parallel sections of the human stomach antrum (Fig. 3). Both antibodies revealed strong positive immunoreactions in the surface epithelial cells. The control MAb showed slightly stronger reactivity in the glands and lamina propria. All these results clearly demonstrated that the 18-9 MAb is specific for S100P protein and approved its use for the immunohistochemical analysis of S100P expression in human tissues (Figs 4 and 5) and carcinomas including breast (Fig. 6A,B), gastric (Fig. 6C), pancreatic (Fig. 6D), ovarian (Fig. 6E,F), prostate, and colon (Fig. 7) tumors. Immunohistochemical results indicated that the placenta indeed expresses very high levels of S100P protein in trophoblast cells (Fig. 4A,B). In the placenta as well as in all other positive tissues, the immunoreactions were strongest in the nuclei and weaker intracellular staining was also detectable. The normal gastric epithelium was another site which showed high immunoreactivity for S100P protein (Fig. 4C,D). In the body of the stomach, the immunostaining was localized to the mucus producing surface epithelial cells and chief cells of the gastric glands. Intestinal metaplasia was occasionally observed in some specimens of the gastric mucosa. The normal epithelium showed stronger immunostaining than the metaplastic epithelium (Fig. 4E). Esophageal mucosa showed immunopositivity which was mainly located to the most superficial epithelial cells (Fig. 4F). The pancreas and liver were mainly negative for S100P (Fig. 2G,H). In these tissues, some positive cell nuclei were occasionally associated with blood vessels, and by histological examination these cells were identified as granulocytes.

**Figure 8:**
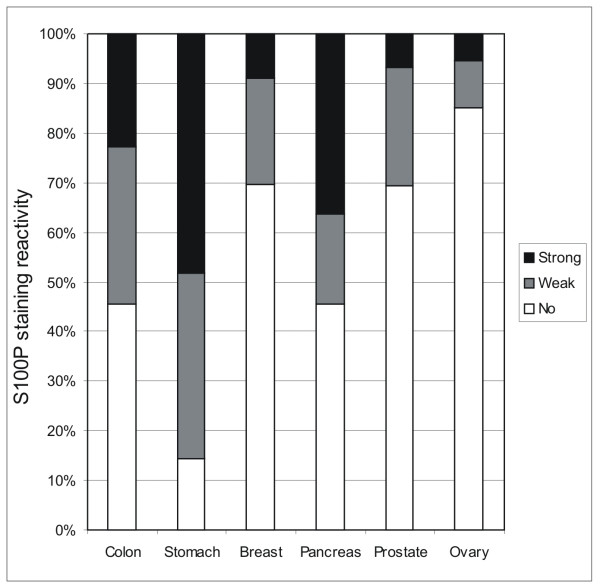
Summary of the immunostaining results in colon, gastric, breast, pancreatic, prostate and ovarian carcinomas.

Various segments of the intestine were also dissected for a more detailed immunohistochemical analysis. The results indicated that all segments of the large intestine including cecum, colon and rectum (data not shown) were positive (Fig. 5C,D). The highest immunoreactions were localized in the epithelial cells of the superficial epithelial cuff region, while the reactions became weaker toward the crypts. In the duodenum, positively stained nuclei were quite frequently present in the enterocytes (Fig. 5A), and positive reactions were also detectable in the jejunum and ileum (Fig. 5B) which showed very low mRNA levels (Fig. 1A). Both lymphoid follicles and smooth muscle of the intestinal wall were negative (Fig. 5E,F). Figure 6 demonstrates S100P protein expression in different malignant tumors including breast, gastric, pancreatic and ovarian carcinomas. Figure 7 shows the staining results in some colorectal carcinomas and benign specimens obtained from the same patients. These figures indicate that the staining reactivity was higher in most tumors (54%) compared to the adjacent benign tissue. It also varied within each tumor category, which is demonstrated in breast (Fig. 6A,B) and ovarian (Fig. 6E,F) carcinomas. The immunostaining results are summarized in Figure 8. Gastric carcinomas showed the strongest immunoreactions, almost half of the specimens having strong signals. From these tumors, the lowest staining reactions were observed in breast, prostate and ovarian cancers. In the tumor categories studied, the expression of S100P showed no correlation to the tumor grade (data not shown).

## Discussion

S100P protein has been recently considered a potential biomarker of cancer due to its frequent expression in different types of tumor tissues [18,19]. Moreover, its direct implication in cancer biology has been proposed on the basis of the experimental data obtained with S100P-transfected tumor cells *in vitro *and *in vivo *as well as of the data from various gene array studies [8,9,15,20-22].

In the present study, we used a newly generated anti-S100P monoclonal antibody 18-9 for immunohistochemical analysis of S100P expression in a series of normal and tumor human tissues. The comparative analysis of the new 18-9 MAb and the commercial control MAb confirmed that these two MAbs are both specific for S100P protein, but bind to different epitopes (data not shown). The immunoreactions obtained with two different S100P antibodies in parallel sections showed that the MAb 18-9 and the commercially available antibody recognize the same cell types in most cases and the subcellular distribution of the immunoreaction is similar. Both antibodies showed the highest immunoreaction in the cell nuclei. In addition, positive intracellular reactions were observed, which most probably reflects either the newly produced protein in the cytosolic compartment of the cell or the fraction of S100P molecules that interact with F-actin-binding protein ezrin [23]. The main difference between these antibodies was detectable in the glands of the gastric mucosa, which seemed to produce slightly higher background staining by the commercial antibody.

Based on the immunostaining and quantitative RT-PCR results, the placenta clearly showed the most prominent expression among all normal tissues. The second most prominent immunostaining was observed in the gastric mucosa. For an unknown reason the mRNA levels in the stomach were comparable to several other segments of the gastrointestinal canal and distinctly lower than in the esophagus which repeatedly showed high mRNA signals and relatively weak or moderate immunohistochemical staining. These inconsistencies between the mRNA and protein levels might reflect tissue type-related differences in secretion of S100P protein to extracellular space, where it appears to function as a signalling molecule via activation of RAGE receptor [24]. Alternatively, they may suggest tissue-specific variations in posttranscriptional regulation of S100P expression or in turnover of S100P protein.

Structural studies have shown that S100P protein exists as a dimer and the S100P homodimer is probably more stable than those of other S100 proteins [25]. High stability is a prerequisite for a good biomarker. In this respect, S100P gene or protein expression has already been proved to correlate with patient survival in lung [7,26] and breast cancer [15], and it has been proposed as an early developmental marker of pancreatic carcinogenesis [19]. Our present results using a newly generated monoclonal S100P antibody confirmed the expression of S100P protein in several tumor categories. It is noteworthy, however, that S100P is not restricted to neoplastic cells, but is also detectable in various normal cell types. This fact has to be carefully considered when planning novel diagnostic and therapeutic applications based on S100P expression.

## Conclusion

The present immunohistochemical and quantitative RT-PCR results demonstrate that S100P protein is widely expressed in both normal and neoplastic tissues. It clearly shows ectopic expression in some cancers. Based on the high expression in certain tumors, S100P could represent a potential target for novel diagnostic and therapeutic applications.

## Competing interests

The author(s) declare that they have no competing interests.

## Authors' contributions

SPar participated in the design of the study, tissue processing, immunohistochemical staining and light microscopy and drafted the first version of the manuscript. PP participated in real-time PCR. AW participated in real-time PCR and immunohistochemistry. AG, IO, SPas and JP participated in the design of the study and produced and characterized the monoclonal S100P antibody. ARM participated in real-time PCR. HOH participated in immunohistochemical staining. JI participated in the design of the study, tissue processing, immunohistochemical staining and light microscopy. All authors helped to draft the manuscript. All authors read and approved the final manuscript.

## Pre-publication history

The pre-publication history for this paper can be accessed here:


